# Beyond trauma: knowledge and training gaps among mental health professionals in the aftermath of October 7th 2023

**DOI:** 10.1186/s13584-025-00734-z

**Published:** 2025-11-27

**Authors:** Or Keynan, Dana Elberg, Shlomo Mendlovic, Ido Lurie, Doron Amsalem, Yuval Neria, Yossi Levi-Belz, Milton Wainberg, David Roe, Asala Halaj, Dana Tzur Bitan

**Affiliations:** 1 Department of Community Mental Health, Faculty of Social Welfare and Health Sciences, University of Haifa, Mount Carmel, 31905 Haifa, Israel; 2 Shalvata Mental Health Center, Hod Hasharon, Israel; 3 School of Medicine, Tel Aviv University, Tel Aviv, Israel; 4 New York State Psychiatric Institute, New York, NY USA; 5 Department of Psychiatry, Columbia University Irving Medical Center, New York, NY USA; 6 The Lior Tsfaty Center for Suicide and Mental Pain Studies, University of Haifa, Haifa, Israel

**Keywords:** Posttraumatic stress disorder (PTSD), Trauma training, Moral injury, Treatment monitoring, Personalized care, Knowledge assessment

## Abstract

**Background:**

Israel experienced a sharp increase in the prevalence of trauma-related disorders after October 7th 2023. Although efforts have been made to train mental health professionals in trauma-related care, it is not clear whether this training is sufficient to address the multifaced and complex needs of patients inflicted by trauma. This study aimed to assess mental health professionals’ subjective experience of trauma-related knowledge as well as their training needs across trauma-related domains.

**Methods:**

Mental health professionals (N = 264) completed an online survey assessing their perceived knowledge of trauma-related diagnoses and their training priorities for trauma-related treatment. The sample included psychologists, social workers, art therapists, and psychiatrists working in the public and private sectors.

**Results:**

Mental health professionals were more knowledgeable regarding posttraumatic stress disorder (PTSD, MD = 0.98 to 2.27, *p* < .001), however, reported significantly greater needs for training focusing on treatment selection for PTSD (MD = 0.41 to 0.89, *p* ≤ .01), on routine outcome monitoring (MD = 0.56 to 0.91, *p* ≤ .005), and on identifying predictors of PTSD prognosis (MD = 0.40 to 0.88, *p* ≤ .02). Participation in trauma-based training moderated the differences in reported knowledge (F = 2.75, p = 0.023, η^2^ = 0.11), with past training leading to greater subjective knowledge in most trauma-related domains (MD 0.27 to 0.81, p ≤ 0.02).

**Conclusion:**

Mental health professionals report of knowledge gaps regarding personalizing treatment approach to PTSD manifestations, as well as treatment management and prognosis. Decision-makers should focus training efforts on treatment personalization and routine outcome monitoring to improve the quality of mental healthcare in Israel.

## Introduction

Many countries inflicted by trauma engage in extensive training for mental health professionals (MHPs) to respond to the sudden increase in mental health needs of citizens experiencing ongoing traumatic distress. This pattern has been observed in many counties undergoing mass traumatic events such as September 11 attack in the US and Russia’s invasion to Ukraine [1–5]. The term ‘collective trauma’ is often used to denote such life-changing situations, whereby a profoundly disruptive event occurring to a group of people creates a shared unresolved loss and vulnerability [6] affecting both the individual and the broader community [7, 8]. Studies suggest that mental health response to collective trauma involves many unique professional demands, including the expansion of workforce [9, 10], the supervision of therapists [10–12] and the provision of tailored training [12]. In Israel, October 7th 2023 attack and the subsequent wars have led to unprecedented increase in mental health needs, with approximately a third of the Israeli population exhibiting symptoms for probable PTSD, with half of the population reporting symptoms of probable depression and anxiety [13]. A recent longitudinal study further demonstrated that rates of PTSD, anxiety and depression continue to be elevated, with over 65% of individuals living in conflict zones reporting heightened symptoms of anxiety, depression or PTSD [14].

Training efforts in Israel have thus far focused on PTSD-informed treatments to professionals responding to the increasing mental health needs after October 7th 2023 [15, 16]. Although these training programs seem to increase accessibility to mental healthcare, it’s suitability to current needs of patients, as perceived by the professionals treating them, has not been systematically evaluated. Studies illustrate that people in Israel had different responses to October 7th 2023 attack and subsequent ongoing conflicts, with some people experiencing other clinical manifestations than PTSD, such as grief [17], suicidal ideation [18] and moral injury [19]. Nonetheless, it is not clear whether professionals in Israel feel sufficiently trained in these clinical manifestations, and whether they are sufficiently knowledgeable in PTSD management to expand their training to other clinical aspects. Conducting such an in-depth assessment of MHP’s subjective experiences is especially warranted seeing that professionals’ competency is considered a predictive factor of treatment success [20, 21]. Furthermore, such systematic evaluation can inform future training initiative and subsequently facilitate a more accurate therapeutic care in times of national crisis, as well as in routine clinical practice.

The present study aimed to investigate MHPs perspectives about their knowledge in trauma care, their training needs, and the moderating impact of past trauma training on these two broad domains. The following research hypothesis were explored: (1) MHPs would report lower perceived knowledge in protocolized treatments and higher unmet training needs in less traditionally emphasized areas (e.g., moral injury, complex grief, and trauma management) compared with protocol-based treatments; (2) previous trauma-focused training will be associated with greater competence and fewer expressed training gaps. The study was conducted as part of the Homiya working group initiative to draft guidelines for trauma-care in Israel.

## Methods

### Participants

Participants were recruited through social media and professional mailing lists. The inclusion criteria were being a MHP providing clinical healthcare and having a sufficient understanding of the Hebrew language. Overall, 529 individuals accessed the online survey and provided informed consent. Of them, 256 (48.39%) did not complete the survey measures. An additional nine participants did not complete at least 50% of the items and were therefore excluded, thus resulting in a total sample size of 264 participants.

Table 1 summarizes the participants’ professional characteristics. The largest group of participants were psychologists (46.97%), followed by social workers (22.73%), art therapists (12.12%), and psychiatrists (9.47%). Most (59.85%) worked both in the public and in the private sector, while 24.24% worked exclusively in the public sector, and 13.64% in the private sector. About 40% reported they were employed in hospitals, with 18.56% employed in HMOs, and 33.71% in other settings. Most of the sample was comprised of specialists (75.38%), and 14.77% identified as interns. About 53% reported having more than 10 years of experience, 24.24% had 5 to 10 years of experience, and 20.45% reported having 0 to 5 years of professional experience. Almost half of the sample reported they had experience with treating trauma-inflicted patients (48.48%), and more than half the sample reported they were trained in protocol-based treatment for trauma (57.20%). Most of the sample (~ 66%) reported they had no experience or use of routine outcome monitoring for clinical practice.

**Table 1:** Professional characteristics of the study sample (*N* = 264)

Factor	*N*	%
Profession		
Psychologist	124	46.97%
Social worker	60	22.73%
Art therapists	32	12.12%
Psychiatrist	25	9.47%
Other	18	6.82%
No response	5	1.89%
Sector of work		
Public	64	24.24%
Private	36	13.64%
Both	158	59.85%
No response	6	2.27%
Place of work		
Health maintenance organization	49	18.56%
Hospital	112	42.42%
Other	89	33.71%
No response	14	5.30%
Level of expertise		
Intern	39	14.77%
Specialist	199	75.38%
Missing values	26	9.85%
Years of experience		
0–5 years	54	20.45%
5–10 years	64	24.24%
10 and above	141	53.41%
No response	5	1.89%
Treating trauma related population	128	48.48%
No response	5	1.89%
Past training in protocol treatment for trauma	151	57.20%
No response	5	1.89%
Routine use in clinical questionnaires	89	33.71%
No response	5	1.89%

### Procedure

The study received the approval of the Institutional Review Board (IRB) of Shalvata Mental Health Center (reference: 0024–24-SHA). An online survey was distributed during December 2024—January 2025 via healthcare mailing lists and social media (Facebook, WhatsApp). The survey was administered through the Qualtrics platform. Participants were asked to electronically sign informed consent and continued the survey only after consenting to take part in the study. After completing the assessments of the study variables, participants were requested to report about their professional identity.

### Measures

An online measure was developed to assess MHPs’ current knowledge about trauma-related conditions, as well as their perceived needs for future training in trauma related care. The measure was developed through a discussion between expert clinicians and researchers (authors SM, YN, IL, YLB and DTB) and included 26 items. These items tapped into three specific domains: knowledge regarding trauma-related conditions, needs for further trauma-related training, and professional characteristics. The first section included seven items assessing familiarity with trauma-related diagnoses and conditions (e.g., "please rate the extent to which you feel knowledgeable about the DSM-5 criteria for PTSD” "please rate the extent to which you feel knowledgeable about diagnosis of prolonged grief"). All items in this section were rated on a six-point Likert chart, ranging from 1 (not familiar at all) to 6 (highly familiar). The second section comprised eleven items evaluating the need for further trauma-related training across a variety of treatment protocols, including prolonged exposure (PE), eye movement desensitization and reprocessing (EMDR), interpersonal psychotherapy (IPT), cognitive processing therapy (CPT) and acceptance and commitment therapy (ACT, e.g., "please rate the extent to which you would benefit from training in ACT treatment technique") as well as by treatment aids (e.g., “please rate the extent to which you would benefit from training on monitoring PTSD treatments"). Items in this section were similarly rated on a six-point Likert chart, ranging from 1 (no need at all) to 6 (essential to the point of impacting my work). The final section included eight questions on professional characteristics (e.g., "What is your profession?", "What is your primary therapeutic orientation?"). Items inquiring about professional characteristics were rated using either yes/no questions or multiple-choice selections. The internal consistency of the knowledge section was sound (α = 0.77) and good for the training section (α = 0.84).

### Statistical analysis

Descriptive statistics were reported using means (M) and standard deviations (SD). Hypothesis 1, examining MHPs perceived knowledge and training needs was examined through two Repeated Measure analyses of variance (ANOVA) models, whereby specific knowledge and training needs served as the within-subject variable. Hypothesis 2 was examined by a mixed-model AVONA, whereby previous knowledge served as the between-subject variable. The Mauchly’s Test of Sphericity was used to examine statistical assumptions of the models. When the assumption of sphericity was violated, the Greenhouse–Geisser correction was applied to adjust the degrees of freedom. Post hoc pairwise comparisons were conducted to identify specific group differences, these differences were reported as mean difference (MD). Multiple comparisons were adjusted for using Bonferroni correction. To examine the moderating effect of past protocol-based treatment training on knowledge and training needs, a mixed-model ANOVA was conducted across items of both exploration fields. All statistical analysis was preformed using SPSS software, version 27.0 (SPSS, Chicago, IL, U.S.A).

## Results

### MHPs’ reported knowledge about trauma related diagnoses

Table 2 presents the means and SDs of reported trauma-related knowledge, organized from highly knowledgeable fields to the lowest, and with comparison markers to denote significant differences. The within-subjects effect of knowledge was statistically significant**, ***F*(4.36, 1116.53) = 179.05, *p* < 0.001, partial η^2^ = 0.41 (Greenhouse–Geisser corrected), indicating significant differences in levels of knowledge in different knowledge fields. Pairwise comparisons were adjusted using the Bonferroni method.

**Table 2:** MHPs’ self-reported trauma related knowledge (N = 264)

Past knowledge in the field of		*M*	*SD*	Items with significantly lower scores
1	Use of structured evaluation	5.29	1.04	3–7
2	Differentiating between trauma exposure and PTSD	5.23	0.87	3–7
3	DSM-5 criteria for PTSD	4.71	1.34	1,2, 4–7
4	Prolonged grief diagnosis	3.73	1.34	1–3, 6, 7
5	Suicidal risk assessment	3.68	1.74	1–3, 6, 7
6	Moral injury	3.19	1.55	1–5, 7
7	Differentiating between potential moral injurious event and MI	2.97	1.63	1–6

Mean levels are obtained from a scale ranging from 1 (‘not knowledgeable at all’) to 6 (‘highly knowledgeable’). Numbers within the column "Items with significantly lower scores" represent the item numbers producing significantly lower scores. For example, for item 1 ‘Use of structured evaluation’, items 3–7 had significantly lower scores as compared to item 1. The within-subjects effect for trauma-related knowledge was statistically significant, F(4.36, 1116.53) = 179.05, p < 0.001, partial η^2^ = 0.41 (Greenhouse–Geisser corrected). Pairwise comparisons are Bonferroni-adjusted

As can be seen, knowledge about diagnostic criteria for PTSD was significantly greater than knowledge about MI (MD = 1.52, SE = 0.11, *p* < 0.001), prolonged grief (MD = 0.98, SE = 0.09, *p* < 0.001), and suicidal risk assessment (MD = 1.03, SE = 0.11, *p* < 0.001), including ‘differentiating trauma exposure and PTSD’, which was significantly higher compared to ‘differentiating potential moral injurious event and MI’ (MD = 2.27, SE = 0.09, *p* < 0.001). Knowledge about identification of MI was also reported significantly lower than suicidal risk assessment (MD = − 0.49, SE = 0.12, *p* = *0.0*02) and prolonged grief diagnosis (MD = − 0.54, SE = 0.09, *p* < 0.001). Furthermore, ‘differentiating between potential moral injurious event and MI’ was significantly lower than knowledge regarding the use of structured evaluation, with a mean difference of −2.32 (SE = 0.11, *p* < 0.001). MHPs reported greater knowledge regarding the use of structured evaluation compared to other categories such as suicidal risk assessment (MD = 1.61, SE = 0.12, *p* < 0.001), prolonged grief diagnosis (MD = 1.56, SE = 0.09, *p* < 0.001), and diagnostic criteria for PTSD (MD = 0.58, SE = 0.10, *p* < 0.001).

### MHPs’ reported needs for further training

Table 3 presents the M’s and SDs of ratings of professional needs in the fields of trauma-related training, organized from highly needed training fields to the lowest, and with comparison markers to denote significant differences. The results indicated statistically significant differences in needs for training, *F*(7.4, 1608.97) = 19.29, *p* < 0.001, partial *η*^*2*^ = 0.08 (Greenhouse–Geisser corrected). Pairwise comparisons were adjusted using the Bonferroni method.

**Table 3:** MHPs’ needs for additional training (N = 264)

Training needs		*M*	*SD*	Items with significantly lower scores
Training in protocol-based treatments				
1	EMDR	3.65	1.33	8–11
2	PE	3.52	1.34	6, 8–11
3	IPT	3.46	1.54	6, 8–11
4	CPT	3.29	1.44	5,6, 8–11
5	ACT	3.78	1.41	4, 9–11
Training in treatment of other diagnostic fields				
6	Prolonged grief treatment	4.00	1.25	2–4, 7
7	Moral injury	3.61	1.42	6, 8–11
8	Suicidal risk treatment	4.16	1.35	1–4, 7
Training in supportive tools to improve trauma therapy outcome				
9	Factors predicting PTSD prognosis	4.18	1.28	1–5, 7
10	Parameters influencing PTSD treatment choice	4.19	1.29	1–5, 7
11	Monitoring of PTSD patients	4.21	1.38	1–5, 7

Mean levels are obtained from a scale ranging from 1 (‘no need at all’) to 6 (‘essential to the point of impacting my work’). Numbers within the column "Items with significantly lower scores" represent the item numbers producing significantly lower scores. For example, for EMDR, items 8–11 had significantly lower scores as compared to item 1. The within-subject effect of needs for training was statistically significant F(7.4, 1608.97) = 19.29, p < 0.001, partial η^2^ = 0.08 (Greenhouse–Geisser corrected). Pairwise comparisons were adjusted using the Bonferroni method. EMDR = Eye Movement Desensitization and Reprocessing; PE = Prolonged Exposure; IPT = Interpersonal Psychotherapy; CPT = Cognitive Processing Therapy; ACT = Acceptance and commitment therapy

**Fig. 1:**
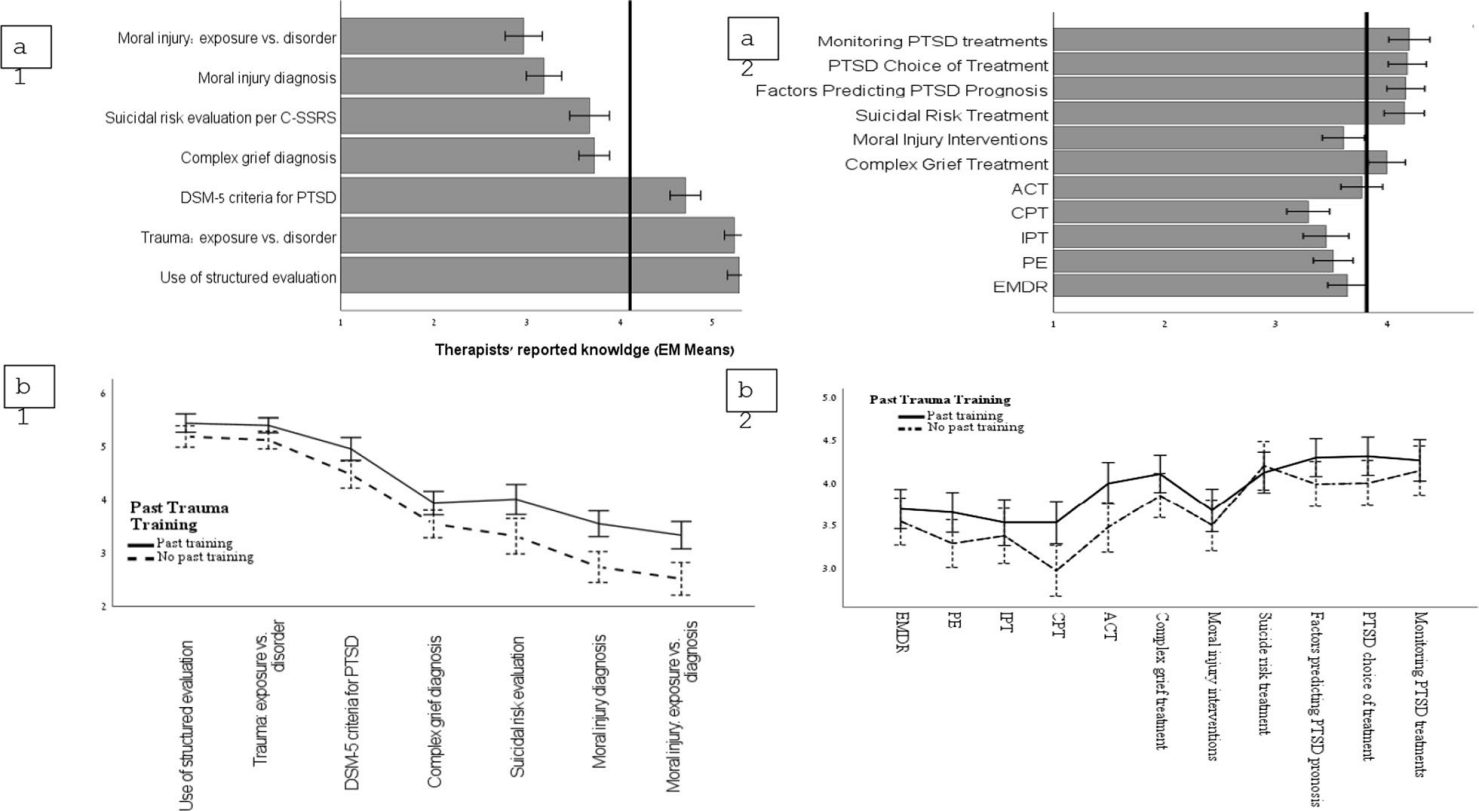
**a** Graphical illustration of MHP’s trauma-related knowledge (left, a1) and training needs (right, a2). **b** Interactive effects of previous training on MHP’s knowledge (left, b1) and training needs (right, b2)

Training regarding PTSD choice of treatment was scored higher compared to training in PTSD protocols such as EMDR (MD = 0.54, SE = 0.11, *p* < 0.001), PE (MD = 0.67, SE = 0.11, *p* < 0.001), IPT (MD = 0.73, SE = 0.11, *p* < 0.001), CPT (MD = 0.89, SE = 0.11, *p* < 0.001) and ACT (MD = 0.41, SE = 0.11, *p* = 0.01). Similarly, the need for training about parameters predicting PTSD prognosis was reported higher compared to all protocolized treatments (MD ranging from 0.40 to 0.88, *p* < 0.001). MHPs also reported higher need in training focusing on monitoring trauma treatments compared to training for each of the protocol-based treatments for PTSD (MD ranging from 0.43 to 0.91, *p* < 0.01). A greater need was also expressed for training in suicidal risk treatments compared to most protocol-based treatments for PTSD (MD ranging from 0.51 to 0.87, *p* ≤ 0.002). The same trend emerged for training regarding prolonged grief treatment, which was scored higher than the need for protocol-based treatments for PTSD training (MD ranging from 0.48 to 0.71, *p* < 0.001). On the other hand, MHPs reported a lower need in training regarding MI treatment compared to prolonged grief, suicidal risk, monitoring of trauma treatments, and PTSD related knowledge (MD ranging from 0.39 to 0.59, p < 0.001). Fig. 1 provides a graphical illustration of the main trends. Thus, MHPs reported lower need for additional training in PTSD treatment protocols but markedly greater demand for skills related to treatment management (e.g., monitoring and prognosis) and for addressing comorbid trauma manifestations such as suicidal risk and prolonged grief.

### The Moderating effects of past protocol-based training

To examine the moderating effect of past protocol-based treatment training on knowledge and training needs, a mixed-model ANOVA was conducted across items of both exploration fields. Table 4 presents the M’s, SDs and significance of these interactive effects.

**Table 4:** Pairwise comparisons of MHPs’ reported knowledge based on past training in protocol treatment for trauma (N = 264)

		Past training (Mean)	No past training (Mean)	Mean difference	SE	95% CI	p
Reported knowledge							
1	Use of structured evaluation	5.39	5.14	0.25	0.13	−0.01, 0.51	0.06
2	Differentiating between trauma exposure and PTSD	5.35	5.08	0.27	0.11	0.06, 0.49	0.01
3	DSM-5 criteria for PTSD	4.91	4.43	0.48	0.17	0.15, 0.81	0.005
4	Prolonged grief diagnosis	3.90	3.51	0.39	0.17	0.05, 0.73	0.02
5	Suicidal risk assessment	3.97	3.28	0.69	0.22	0.25, 1.12	0.002
6	Moral injury	3.51	2.70	0.81	0.19	0.44, 1.19	< 0.001
7	Differentiating between potential moral injurious event and MI	3.30	2.48	0.82	0.20	0.42, 1.21	< 0.001
Reported training needs							
1	EMDR	3.70	3.56	0.14	0.18	−0.22, 0.51	0.43
2	PE	3.66	3.30	0.36	0.19	0.00, 0.74	0.05
3	IPT	3.54	3.39	0.15	0.22	−0.27, 0.58	0.46
4	CPT	3.54	2.98	0.56	0.20	0.18, 0.96	0.005
5	ACT	4.01	3.49	0.52	0.19	0.14, 0.90	0.008
6	Prolonged grief treatment	4.12	3.86	0.26	0.17	−0.09, 0.60	0.14
7	Moral injury treatment	3.69	3.51	0.18	0.20	−0.22, 0.57	0.38
8	Suicidal risk treatment	4.14	4.22	−0.08	0.19	−0.45, 0.29	0.67
9	Factors predicting PTSD prognosis	4.31	4.00	0.31	0.18	−0.04, 0.66	0.08
10	PTSD choice of treatment	4.33	4.01	0.32	0.18	−0.03, 0.67	0.08
11	Monitoring PTSD treatments	4.28	4.16	0.12	0.19	−0.26, 0.50	0.53

For knowledge, a significant within-subjects effect was found for the knowledge domain, F(4.37, 1092.13) = 179.29, p = 0.023, partial η^2^ = 0.42, indicating variation in knowledge across trauma-related fields. The between-subjects main effect of past training was not significant, F(1, 250) = 3.85, p = 0.051, partial η^2^ = 0.02, suggesting a non-significant difference in overall knowledge across trained and non-trained clinicians. The interaction effect between past training and knowledge was statistically significant**, ***F*(4.37, 1092.13) = 2.75, *p* = 0.023, *η*^*2*^ = 0.11. Post hoc pairwise comparisons indicated that MHPs with past protocol training demonstrated significantly higher knowledge levels compared to those without training in most of the knowledge domains, including knowledge related to PTSD (trauma exposure and PTSD, diagnostic criteria for PTSD, and other conditions (such as prolonged grief and suicidal risk) but not in knowledge regarding the use of structured evaluation (MD = 0.25, SE = 0.13, *p* = 0.06). For training needs, there was a significant within-subjects effect of training domain F(7.49, 1581.17) = 19.42, *p* < 0.001, partial η^2^ = 0.084), indicating that perceived training needs differed across the examined domains. The between-subject effect of past training was significant as well F(1, 211) = 4.98, *p* = 0.03, partial η^2^ = 0.023), suggesting that clinicians with prior training reported overall lower training needs than those without such training. No significant interaction effect was detected in training needs, (*F*(7.49, 1581.17) = 1.40, *p* = 0.20, *η*^*2*^ = 0.007), thus indicating that, overall, trained MHPs have the same needs as those who did not undergo protocolized based training.

## Discussion

The present study aimed to identify MHPs’ subjective experience of knowledge about trauma treatments in the aftermath of October 7th 2023, as well as their professional training needs. The results indicated that MHPs feel knowledgable regarding PTSD compared to other trauma-related conditions such as MI, prolonged grief, and suicidal risk. Furthermore, MHP’s were found to have less needs for training in protocol-based PTSD treatments, and more needs for training in other trauma-related conditions, as well as more needs for treatment management such as personalizing treatment to patients’ needs and performing routine assessment of outcomes. MHPs who previously participated in training reported higher knowledge in most assessed fields of knowledge, however, their professional needs were the same as those without prior training.

The results of the current study suggests that while MHPs are sufficiently knowledgable regarding PTSD, they are less knowledgable about other clinical menifistations of trauma, such as prolonged grief and MI. As in other mass truamtic events, most training programs initiated following October 7th 2023 focused on PTSD and its diagnostic creteria [16]. Nonetheless, trauma has many clinical facets, including suicide ideation [18], prolonged grief [17], and MI [19], with national findings following October 7th 2023 indicating significant increase in MI symptoms, as well as grief responses which are often interwined [17]. Taken together, these findings suggest that focus should shift from protocol-based PTSD treatments, to training of other comorbid conditions such as prologed and complex grief, suicidal ideation and MI.

Reported gaps in knowledge were also ecoed in MHP’s subjective report of their training needs. MHPs reported greater demand for training in suicial risk interventions and the management of grief, both clinical areas with well validated, evidence-based protocols which can be readlily adopted and implemented [22]. Although reports regarding suicidal ideation in Israel have been incosistent, MHP’s training needs regarding management of suicidal patients may be related to reports about an increase in suicidal ideation after October 7th 2023 [18]. Studies indicate that war and terror related trauma are closely associated with suicidal ideation, with each additional trauma associated with an increase of 20.1% in rate of suicidal ideation and 38.9% in rate of suicide attempts [23]. Thus, the management of grief and working with suicidal patients may constitue a priority in future initiatives aim to train professionals in trauma-related conditions.

The most highly ranked items for training needs, as reported by the MHPs, were training in monitoring trauma treatments, and personalizing the therapeutic modality to patients’ needs. Studies conducted in Israel and worldwide have demonstrated that routine outcome monitoring (ROM) improves therapeutic outcomes and patient-therapist alliance, with benefits extending beyond diagnostic criteria or treatment modality [24–27]. Furthermore, the official clinical treatment guidelines by the U.S. Department of Defense and the Department of Veterans Affairs emphasizes the advantages of using ROM to facilitates early identification of deterioration, adjust therapeutic approaches, and replace ineffective treatments [28]. These recommendations resonate with MHPs’ reported need to learn how to personalize the therapeutic intervention, a step which seems appropriate in cases where patients do not demonstrate sufficient improvement. Overall, these findings suggest that MHPs need more tools to manage the course of treatment, rather than being trained for specific interventions.

The findings reported in this study resonate with global research of collective trauma, which demonstrates the tendency to prioritize PTSD while neglecting other trauma-related domains such as prolonged grief, loss, and moral injury [29–31]. To illustrate, Watson et al. [32] reviewed adaptations in mental health care following disater since 9/11 disasters and found a lack of attention to treatment of grief. Similarly, McFarlance et al. [33] reviewed mental health services following diverse large-scale traumas worldwide- including the bushfires in Australia, the London bombings (2005), and the compound disaster in Fukushima, Japan (2011)- and concluded that providers must be prepared to address trauma-related conditions such as grief and suicidality, rather than focusing exclusively on PTSD. The findings of the current study extend these reports by demonstrating that these gaps are not only confined to training programs, but are also reflected in MHPs subjective experience of insufficient preparedness to treat these comorbid conditions.

Several impotrant implications to inform Israel’s national mental health policy can be outlined. Recent initiatives of the Ministry of Health emphasize the need to train professionals on evidence-based therapeutic approaches targeting trauma-related conditions [15]. The results of the study extend this important call to focus not only on PTSD treatment protocols, but also on advanced techniques of treatment management such as measurement-based care and treatment personalization. The findings also support the recent efforts made by the Ministry of Health to focus on suicidality prevention [34–36], while stressing the need to focus training efforts not only on early identification, but also on treatment management of patients with ongoing and persistent suicidality. These important insights may also inform global policy development in countries affected by regional conflicts. Specifically, they underscore the value of a stratified, needs-based approach to training, where education and supervision are tailored to the evolving professional needs of practitioners over time. Furthermore, they highlight the importance of integrating training in trauma-management and treatment adaptation strategies as means to build system-level preparedness in the aftermath of collective trauma. From a theortical perspective, the results also emphasize the need to expand the concept of trauma-informed care beyond PTSD, to acknowledge other trauma related conditions such as prolonged grief, ambiguous loss, and moral injury.

Several steps can be suggested to adapt the findings into actionable policy changes. Governmental incentives aimed to enchorage professional development of licenced practioners should prioritise brief courses which focus on treatment of moral injury, management of suicidal patients, and measurement-based care. Quality measures of the Ministry of Health can prioritize excellance not only in trauma-care but also on treatment management and/or comorbid clinical menifestations of trauma. Academic curriculums can be tailored to meet the public mental health sector training needs, while adapting contents to the specific challenges of Israel’s public mental health system. Previous studies have demonstrated that issues related to insufficient workforce and lack of digitalization impede the ability to fully implement advanced approaches for treatment management such as ROM [26]. Nonetheless, the current climate of expending licensure for mental health practitioners [37], as well as the ongoing recognition of the urgent need to increase accessibility and quality of care [38], may pose an opportunity to focus national efforts on building a strong infrastructure for high-quality mental healthcare in Israel. In this sense, policymakers should consider adopting worldwide recommendations derived from implementation science, which emphasize the integration beween top-down and bottom-up processes to innovation implementation [39]. Alternatively, a tiered implementation model which fits the level of implementation to the clinical unit’s ability to motivate change [40] may gradually allow for innovative techniques to dissiminate in the public mental healthcare domain.

Several limitations should be acknowledged. This study utilized a self-report measure to assess MHPs’ knowledge from their own perspective, rather than through an objective measure of adherence or proficiency in therapeutic protocols. Additional studies are needed to assess whether the increased subjective knowledge also translates to more effective therapy in real-life settings. Additional studies should be performed to fully examine the scale’s psychometric properties, as well as to validate our findings using other, well-validated scales. As a comparison between the dropout and completer sample could not be performed, additional studies are needed to further explore the level of generalizability of our findings. Additional studies are needed to practically investigate viable means to implement novel training approaches which can narrow the reported gaps. Studies conducted in Israel demonstrate cultural and systemic barriers to adopting new therapeutic models particularly in relation to suicide, grief, and ambiguous loss. These include for example inaccessibility of cross-cultural supervision and language issues [41], therapists’ objections and beucratic and organizations issues [25, 26, 42]. Cultural barriers among minority groups such as Israeli Arabs [43] and ultra-Orthodox Jews [44] further include stigma and social inclusion, all likely to severely impede the ability to train and provide adequate care for these populations. These barriers should be further explored. Controlled clinical studies are needed to examine the effectiveness of treatment protocols targeting grief, moral injury, and suicidality, to inform clinical practice. Studies utilizing mix-methods approaches can also be used to further delineate MHPs perspectives about training strategies. Future research may also explore unique psychotherapeutic challenges arising during collective trauma, where clinicians themselves are exposed to the same traumatic distress as their patients [45, 46]^.^

Notwithstanding these limitations, the results of the current study highlight the importance of continuously assessing MHPs’ knowledge and training needs, rather than assuming that a specific training is needed, particularly in times of resource scarcity. Furthermore, they illustrate the necessity of maintaining a flexible and adaptive approach to training, by regularly evaluating whether MHPs possess adequate proficiency in treatment strategies or alternatively require additional training in other fields of interventions. On the national level, the findings suggest that future training strategies for mental health professionals should adopt a more integrative approach to guidance and supervision, while shifting the focus from protocol-based PTSD training to programs that address the co-occurrence of grief and MI, and adapt strategies for managed care.

## Data Availability

Due to ethical restrictions, data will not be publicly available. Aggregated data will be available from the corresponding author.

## Abbreviations

PTSD: Post traumatic stress disorder
MI: Moral injury
MHP: Mental health professionals
HMO: Health maintenance organization
M: Mean
MD: Mean difference
SE: Standard error
SD: Standard deviation
ANOVA: Analysis of variance
EMDR: Eye movement desensitization and reprocessing
PE: Prolonged exposure
IPT: Interpersonal psychotherapy
CPT: Cognitive processing therapy
ACT: Acceptance and commitment therapy
DSM-5: Diagnostic and statistical manual of mental disorders, fifth edition
ROM: Routine outcome monitoring
